# Positron emission tomography combined with serum biomarkers detects fibrotic MASH

**DOI:** 10.1038/s41598-024-72655-x

**Published:** 2024-09-20

**Authors:** Sean Romeo, Connie Chan, Karen Matsukuma, Michael T. Corwin, Victoria Lyo, Shuai Chen, Guobao Wang, Souvik Sarkar

**Affiliations:** 1 Division of Gastroenterology and Hepatology, Department of Internal Medicine, University of California, Davis, CA USA; 2 School of Medicine, University of California, Davis, CA USA; 3 Department of Pathology and Laboratory Medicine, University of California, Davis, CA USA; 4 Department of Radiology, University of California, Davis, CA USA; 5 Department of Surgery, University of California, Davis, CA USA; 6 Division of Biostatistics, Department of Public Health Sciences, University of California, Davis, CA USA

**Keywords:** Metabolic engineering, Non-alcoholic fatty liver disease, Diagnostic markers

## Abstract

Metabolic dysfunction-associated steatohepatitis (MASH) is a rising global disease signaling the urgent need for non-invasive tests (NITs). Recent work demonstrated that dynamic ^18^F-fluorodeoxyglucose (FDG) positron emission tomography (PET)/computed tomography (CT) imaging can identify MASH by measuring liver glucose transport rate, K_1_, and liver CT attenuation. By combining dynamic PET/CT with the serum-based fibrosis-4 (FIB-4) test, we were able to better distinguish clinical MASH from fibrotic subtypes, enabling determination of the core tenets of MASH: steatosis, inflammation, and fibrosis. Future studies using FDG-PET technology can further enable concomitant prediction of MASH severity and extrahepatic comorbidities such as cardiovascular disease.

## Introduction

Metabolic dysfunction-associated steatohepatitis (MASH) is a severe form of metabolic dysfunction-associated steatotic liver disease (MASLD) that is associated with liver cirrhosis and hepatocellular cancer^1^ (MASLD and MASH are new nomenclature for nonalcoholic fatty liver disease, NAFLD and nonalcoholic steatohepatitis, NASH, respectively^2^. Increasing global MASLD prevalence makes early diagnosis of MASLD and MASH important to prevent severe liver damage and extrahepatic disorders possibly related to MASLD such as atherosclerotic cardiovascular disease^3^. MASH is widely underdiagnosed due to its asymptomatic nature and has varying rates of progression, making disease severity gradation difficult. The gold standard for diagnosing MASH is a liver biopsy, but complication risks and patient discomfort present a need for non-invasive methods that can characterize the core tenets of MASH: steatosis, inflammation, and fibrosis^1^. Non-invasive modalities such as an ultrasound-based transient or shear wave elastography have validated data relevant for liver steatosis and fibrosis but not inflammation^4^.MRI-based technique such a MR-PDFF and MR elastography can detect steatosis and fibrotic MASH with excellent accuracy^5^. Advancement in MRI technology has enabled detection of inflammation using damping ratio^6^. These modalities unlike PET are limited by their static nature, and inability to scan other organs simultaneously which is relevant in systemic diseases such as MASH.

Glucose uptake is altered by MASH-related inflammation and hepatic steatosis and can be worsened by tissue fibrosis and collagen deposition as the disease progresses. Positron emission tomography (PET) with the radiotracer ^18^F-fluorodeoxyglucose (FDG) can be used to visually capture glucose kinetics and metabolism. Static PET scans are typically administered to measure glucose analogue’s uptake by the target organ. After the radiotracer is injected, a single time point is captured to show its spatial distribution, thus providing the standardized uptake value (SUV). This technique has been demonstrated in previous studies that measure glucose activity in obese and MASLD patients^3^. The organ or tissue of interest can be further evaluated through dynamic PET imaging, which incorporates a temporal component to the readings by scanning the radiotracer at multiple time points. This data can be further analyzed using tracer kinetic modeling techniques and estimate physiologic parameters including blood flow and transport rates. In the context of the liver, dynamic FDG-PET can be used to elucidate glucose transport by measuring the blood-to-tissue FDG transport rate K_1_^7^, unlike conventional FDG-PET methods that mostly focus on assessing glucose metabolism. Liver inflammation in MASH is pathologically characterized as lobular inflammation and ballooning degeneration – the former is defined by necroinflammatory foci of which the likely cause is increased cell death, and the latter is directly a form of cell death. These cell death processes (e.g., apoptosis, necroptosis) are initiated but not necessarily completed during MASH^8^, and their increase can be linked with decreased glucose transport^9^. Our studies^3,7^ indeed suggested that a lower glucose transport is associated with increased liver inflammation and can be an indicator for MASH-induced inflammation.

The study aims to develop a novel diagnostic model with high sensitivity and specificity in detecting MASH. Similar diagnostic tools have been utilized in oncology and Alzheimer’s research, but none have combined serum and PET imaging scores as a biomarker for disease activity^10–15^. Our previous study found that a dual-variate model combining the dynamic PET measure of liver glucose transport rate K_1_ and liver CT attenuation detected clinical MASH with ample sensitivity and specificity but was unable to detect fibrotic MASH with sufficient specificity^16^. The fibrosis-4 (FIB-4) score is a serum-based biomarker of fibrosis in liver disease^17^. Incorporating patients’ FIB-4 scores accounts for the variance in fibrosis progression in MASH without any additional invasive testing. Herein, we establish a triple variate model that combines PET/CT imaging and serum-based biomarkers, developing a radiologic tool that can reliably elucidate imaging determinants of fibrotic MASH.

## Method

The study was approved by the University of California, Davis institutional review board (IRB). Informed consent was obtained from all participating patients prior to any study procedures. Only consenting patients ≥ 18 years with MASLD who had undergone liver biopsy within 6 months of planned imaging were enrolled. All methods were performed in accordance with the relevant guidelines and regulations. Pregnant patients, prisoners, and patients with a history of alcohol abuse, chronic hepatitis B or C, or other chronic liver disease other than non-alcoholic fatty liver disease were excluded from the study. Liver biopsies were scored per NASH-CRN (Non-alcoholic Steatohepatitis-Clinical Research Network) criteria. Fibrotic MASH was defined as MASLD activity score (NAS) ≥ 4 with Kleiner fibrosis stage ≥ 2. Clinical MASH was defined as MASH ≥ 4 with a score of at least 1 in each of the MASH categories. All patients completed a dynamic ^18^F-FDG PET/CT scan on a GE Discovery 690 scanner within 6 months of liver biopsy^16^. Diabetic patients were instructed to hold any medication used to manage type 2 diabetes mellitus for 24 h leading up to the scan. All patients were required to refrain from any rigorous exercise for 24 h before the scan. Patients received a bolus injection of 10 mCi ^18^F-FDG and dynamic images of the liver were collected over 60 min. A low-dose CT scan was performed at 140 kVp for PET attenuation correction. A time activity curve (TAC) of the liver from the dynamic sequence was extracted by placing eight spherical regions of interest (ROI) in the liver. ROIs were 25 mm in diameter and placed on each of the eight segments of the liver. The TAC was determined by averaging the FDG activity of collective ROIs. Following the same kinetic modeling approach that we developed previously^7,18^, the liver FDG blood-to-tissue transport rate, K_1_, was determined using a reversible two-tissue compartmental model with an optimization-derived dual-blood input function model that accounts for liver dual blood supply from the portal vein and hepatic artery. Liver CT hounsfield units (CTHU) were also measured from the liver ROIs. CT-derived CTHU was selected for MASH evaluation because CTHU has been well validated for liver steatosis^19^, and per protocol low dose CT scan was done at the end of the dynamic scan. With CTHU, CT attenuation of different tissues is measured quantitatively by Hounsfield units (HU) with water assigned a level of 0 HU. Normal liver has attenuation of approximately 55 HU. Fat is less dense than water, and thus has attenuation less than 0, −20 to −100. When fat is present in the liver, it lowers the attenuation of the tissue, often less than 40 HU. The liver CTHU is thus negatively correlated with the degree of steatosis. FIB-4 scores were calculated from age, aspartate aminotransferase (AST), alanine aminotransferase (ALT), and platelet count within 90 days of PET/CT imaging. Correlations among parameters were calculated using the Spearman’s correlation coefficients. Receiver operating characteristics (ROC) analyses were performed with cut-off selected based on Youden’s index, where FIB-4, K_1,_ and CTHU were combined using linear logistic regression with all three variables included in the initial model. We then perform forward variable section by further trying to add their 2-way interactions, where an interaction was added if the akaike information criterion (AIC) of the model was reduced. The final selected model is the initial model (i.e., only including main effects of the three variables without interactions). Data were analyzed using R version 4.0.4 (R Foundation for Statistical Computing, Vienna, Austria).

## Results

**Fig. 1:**
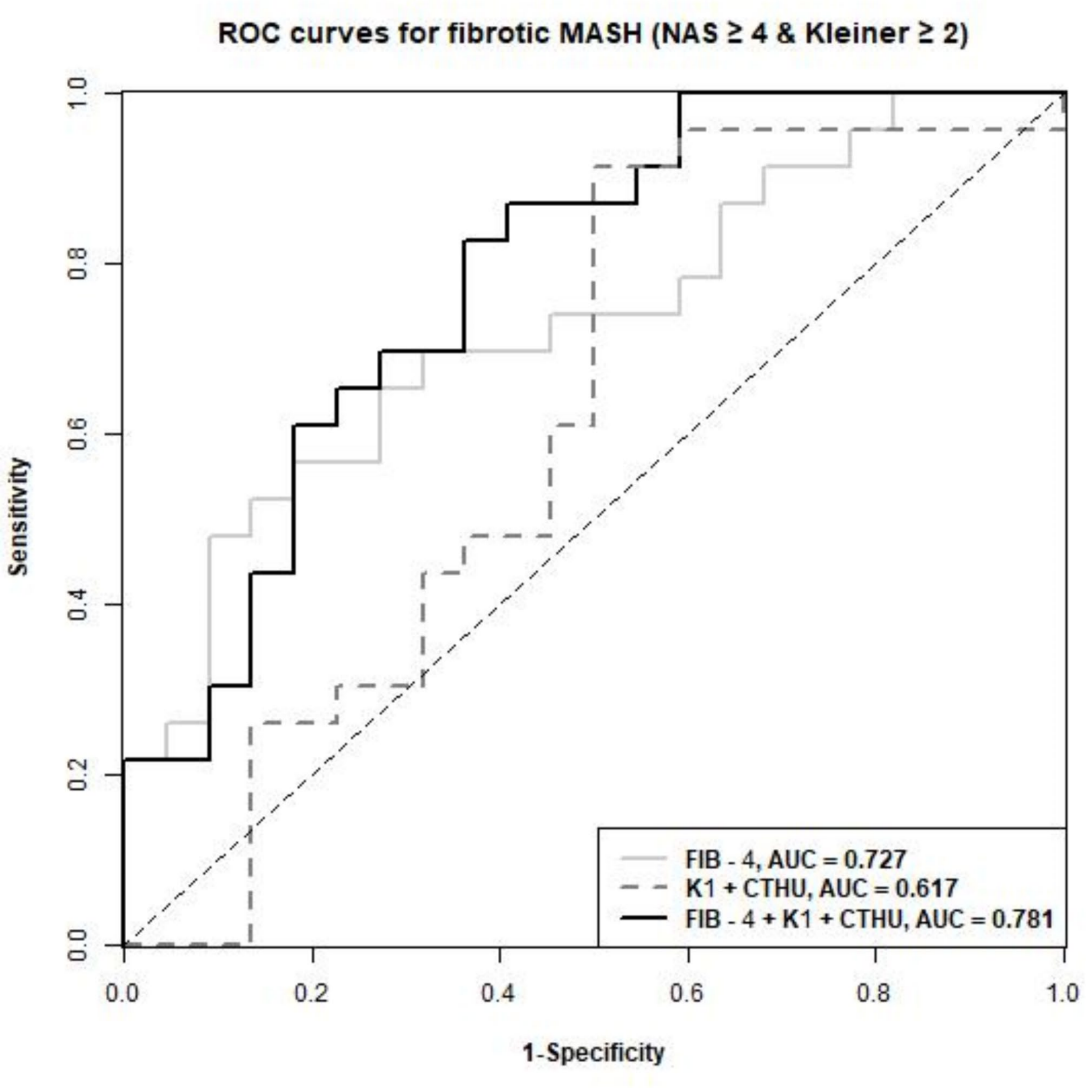
Receiver operating characteristics (ROC) for detecting fibrotic MASH using the triple-variate model in relation to the dual-variate model or FIB-4 alone.

**Fig. 2:**
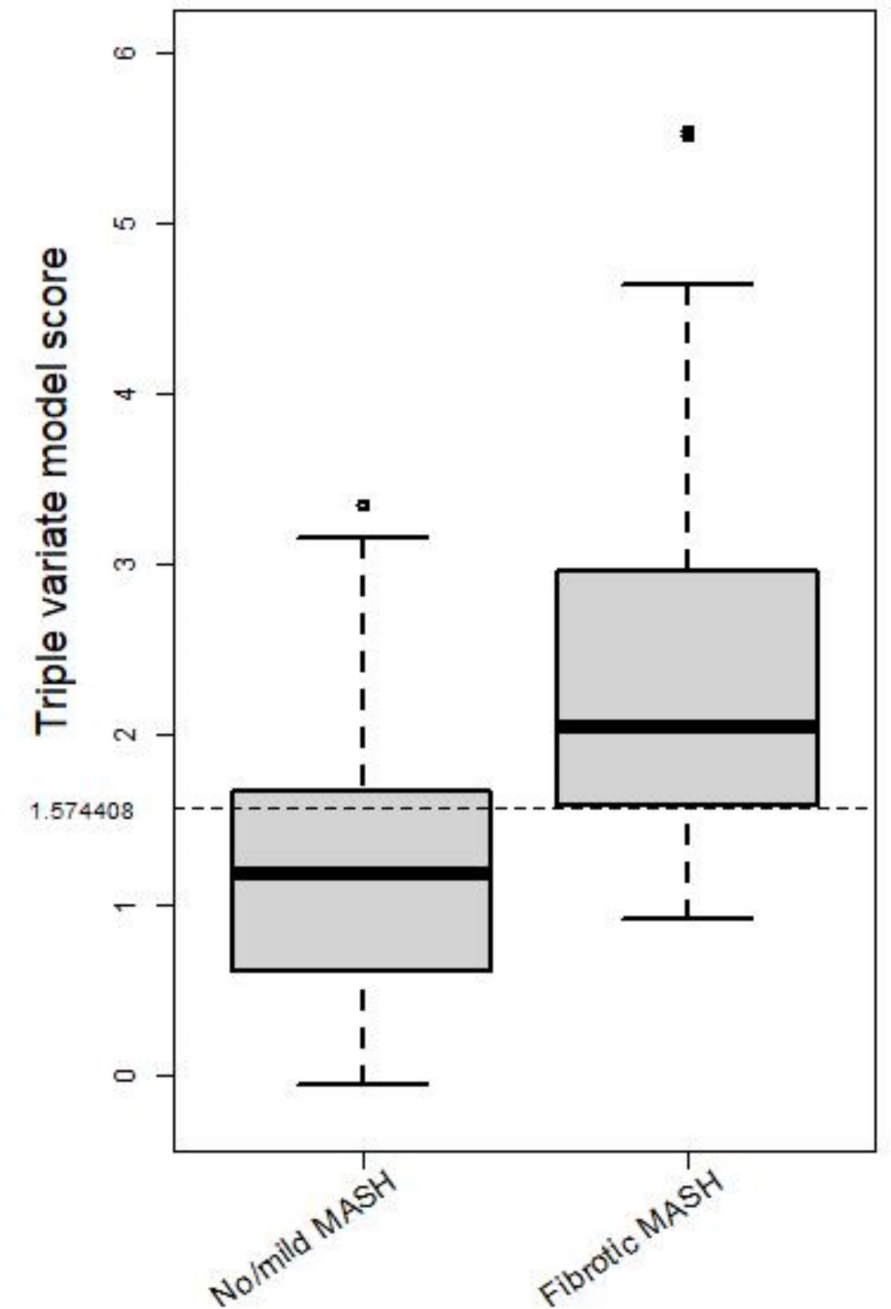
Boxplot of the score from the triple-variate model with a cut-off selected by Youden’s Index, stratified by fibrotic MASH and no/mild MASH groups.

Of 45 enrolled patients, 31 were female, age 54 ± 13 years, 91% Whites, 22% Hispanic or Latinos, 31% with known diabetes, and body mass index of 34.0 ± 5.7 kg/m^2^ (Table 1). 16% of patients had inflammation score > 3, while 82% had NAS ≥ 4 and 56% with fibrosis score ≥ 2. FIB-4 correlated with fibrosis significantly (r = 0.58, p < 0.001), as expected, but not with NAS (r = 0.2, p = 0.178), whereas K_1_ and CTHU showed significant correlations with NAS. As shown previously (9), the dual-variate model of K_1_ + CTHU could detect clinical MASH but not fibrotic MASH. The FIB-4-only AUC was 0.727, but when FIB-4 score was combined in the regression model, it detected fibrotic MASH (NAS ≥ 4, Kleiner fibrosis ≥ 2) with an area under ROC (AUC) of 0.781 (Fig. 1). The triple-variate model of 1.46001*K_1_−0.04009*CTHU + 1.27309*FIB-4 ≥ 1.574408, predicted fibrotic MASH with a sensitivity of 83% and a specificity of 64%. Boxplots show delineation of fibrotic MASH with no/mild MASH (Fig. 2), showing excellent ability to differentiate fibrotic MASH using the triple variate model.

**Table 1:** Patient characteristics.

Characteristic	All (N = 45)	Non-fibrotic MASH (N = 22)	Fibrotic MASH^a^ (N = 23)
	Mean (SD)	Mean (SD)	Mean (SD)
	Median (range)	Median (range)	Median (range)
Age	54.5 (12.7)	52.1 (13.7)	56.7 (11.5)
	56 (18–77)	53 (18–70)	57 (28–77)
BMI	34.0 (5.7)	33.4 (5.0)	34.6 (6.4)
	32.3 (24.0–47.4)	31.4 (25.3–46.0)	33.2 (24.0–47.4)
FIB-4	1.9 (1.4)	1.4 (0.6)	2.4 (1.7)
	1.5 (0.3–8.4)	1.3 (0.3–3.1)	2.0 (0.8–8.4)
K_1_	1.01 (0.21)	1.03 (0.19)	0.99 (0.23)
	0.96 (0.67–1.84)	0.99 (0.72–1.55)	0.95 (0.67–1.84)
CTHU	46.5 (13.2)	48.2 (15.7)	45.0 (10.5)
	49.0 (16.6–67.2)	51.3 (16.6–67.2)	48.8 (26.2–56.4)
	N (%)	N (%)	N (%)
Gender			
Female	31 (68.9)	12 (54.6)	19 (82.6)
Male	14 (31.1)	10 (45.5)	4 (17.4)
Race/ethnicity			
Non-hispanic white	31 (68.9)	16 (72.7)	15 (65.2)
Hispanic	10 (22.2)	5 (22.7)	5 (21.7)
Asian	2 (4.4)	1 (4.6)	1 (4.4)
Unknown	2 (4.4)	0 (0.0)	2 (8.7)

^a^Fibrotic MASH is defined as NAS ≥ 4 and Kleiner ≥ 2.

## Discussion

Our study demonstrates the effectiveness of using dynamic FDG-PET/CT imaging combined with serum-based test FIB-4 as a non-invasive tool to detect fibrotic MASH, complementing studies that use ultrasound or MRI^15,20,21^. Newsome et al. evaluated the FibroScan-AST (FAST) score in identifying MASH patients that combined liver stiffness measurement (LSM) and controlled attenuation parameter (CAP) from the FibroScan® with the lab test aspartate aminotransferase (AST): alanine aminotransferase (AST:ALT) ratio^20^. The FAST test showed a sensitivity of 48%. Jung et al. found that MRI-based MR elastography (MRE) alone demonstrated clinically significant diagnostic accuracy for the detection of fibrotic MASH^15^. However, when MRE was combined with FIB-4 together, Jung et al. found it to have a higher AUROC with a positive predictive value (PPV) of 97.1% but a sensitivity of 56%^15^. While Truong et al. combined AST with MRI sores for steatosis (MR-PDFF) and fibrosis (MR elastography) to give the MAST score that also was effective in delineating fibrotic MASH^21^. MAST also fared well as a prognostic tool for predicting major adverse liver outcomes (MALO)^21^. A critical finding from these studies is that combining serum markers with the imaging biomarkers for MASH improved accuracy and enabled a more holistic approach to diagnosis and risk stratification. Our triple-variate model enabled combination of all the available imaging and biochemical markers relevant to the PET imaging space. Our model complements the other non-invasive methods and provides a sensitive tool for determining fibrotic MASH with a sensitivity of 83%. We will be collecting longer-term data to elucidate the ability of this tool for predicting major adverse liver outcomes (MALO) and extrahepatic outcomes (namely, cardiovascular, renal etc.).

The addition of the FIB-4 index creates a novel triple-variate model that detects a wider range of MASH severity in patients’ diagnoses with adequate sensitivity. This can serve as a noninvasive substitute for liver biopsies and circumvent the use of invasive methods. Furthermore, because the main cause of morbidity in MASH patients remains cardiovascular disease, FDG-PET, which has been used for clinical cardiac imaging, can enable taking a more comprehensive approach in predicting comorbidities affecting the heart or kidney^22–24^. The new PET/CT model will not only detect MASH, but it will also detect increased risk of myocardial impairment via identification of focal myocardial ^18^F-FDG reuptake patterns and assess renal function using multiple positrons emitting radiolabeled racers^22,23^. Lastly, a critical functionality of FDG-PET is in cancer imaging. Pre-determination of liver disease stage in such patients undergoing PET imaging will enable personalizing therapies based on risk of liver toxicity^25^. Increasing the reliability of the PET/CT model will provide physicians with a clinical advantage to address both MASH symptoms and extrahepatic disease and risk factors. We envision the utilization of this technology in a variety of settings. In an initial phase it can have significant applicability where FDG-PET remains prevalent, such as in oncologic settings. It can provide diagnostic capability to determine fibrotic MASH in patients to help choose treatment regimen based on liver risk stratification. For general clinical purposes, we do not foresee our method replacing easily available point-of-care imaging tools such as transient elastography. Detailed imaging modalities such as MRE/MR-PDFF will continue to play a niche role for clinical trials or difficult to diagnose patients. With the significant progress in the PET imaging space especially with newer tracers (e.g. FAPI^26^) and whole body or total-body imaging, we envision our method will have an impact in determining liver disease in the context of systemic diseases such as diabetes and obesity. As the armamentarium of MASH therapies increase especially with treatments that target multiple systems (e.g. Tirzepatide, Semaglutide), it will become increasingly essential to have a tool that can concomitantly evaluate systemic disease. Annual monitoring of response to treatment, not just from the liver standpoint (steatosis, inflammation, and fibrosis) but also overall cardiac and renal disease^24^ will be essential.

The current methodology is intended to establish a technology that can be applied to a widely prevalent disease. Currently, a 60 min dynamic scan would largely be restricted to clinical trial settings in academic medical centers. Advances in technology including improved tracer data capture, and analysis along with machine learning applications that is enabling substantial decrease in dynamic scan protocol to obtain comparable data. Thus, we envision a near future where dynamic PET protocol can be achieved in ~ 15 min that can capture data with high sensitivity relevant for both oncology and metabolic applications. This will make it attractive and relevant to Onco-PET practices and patient care. Although radiation exposure is a risk, effective dose remains low and comparable to abdominal CT^27^. The total effective radiation dose from this PET/CT scan is lower than that of a clinical PET/CT and is accepted to be below the levels thought to result in a significant risk of harmful effects^27^.

Some of the limitations of this study include its small sample size and localization to a single center. Adapting this tool in larger and diverse cohorts will enable establishing this method for determination of clinical changes in MASLD patients. The triple variate model predicted fibrotic MASH with a sensitivity of 83% and specificity of 64%. Liver biopsies are the gold standard, and our triple variate model is not intended as a substitute but rather an improved non-invasive method relative to existing non-invasive methods. With liver biopsies, we can encounter sampling errors, intra-and inter-observer variability^28^; high cost; and adverse effects such as pain, risk of infection, bleeding, perforation, and though rare, potentially death^29^, can deter patients from getting screened for MASH.

In conclusion, the triple variate model provides effective determination of fibrotic MASH, enabling utilization of PET/CT for larger scale studies in MASLD patients.

## Data Availability

Deidentified data may be provided upon reasonable request.
